# Genetic Confirmation of the Role of Sulfopyruvate Decarboxylase in Coenzyme M Biosynthesis in *Methanococcus maripaludis*

**DOI:** 10.1155/2013/185250

**Published:** 2013-09-12

**Authors:** Felipe Sarmiento, Courtney K. Ellison, William B. Whitman

**Affiliations:** Department of Microbiology, University of Georgia, 541 Biological Science Building, Athens, GA 30602-2605, USA

## Abstract

Coenzyme M is an essential coenzyme for methanogenesis. The proposed biosynthetic pathway consists of five steps, of which the fourth step is catalyzed by sulfopyruvate decarboxylase (ComDE). Disruption of the gene *comE* by transposon mutagenesis resulted in a partial coenzyme M auxotroph, which grew poorly in the absence of coenzyme M and retained less than 3% of the wild type level of coenzyme M biosynthesis. Upon coenzyme M addition, normal growth of the mutant was restored. Moreover, complementation of the mutation with the wild type *comE* gene in *trans* restored full growth in the absence of coenzyme M. These results confirm that ComE plays an important role in coenzyme M biosynthesis. The inability to yield a complete CoM auxotroph suggests that either the transposon insertion failed to completely inactivate the gene or *M. maripaludis* possesses a promiscuous activity that partially complemented the mutation.

## 1. Introduction

Hydrogenotrophic methanogens, such as *Methanococcus maripaludis*, possess a specialized metabolism. In a process known as methanogenesis, they reduce CO_2_ to CH_4_ using H_2_ or formate as the electron donor [1]. While other methanoarchaea can use acetate, methylamine, and other methyl-group-containing compounds [1], coenzyme M (CoM), the smallest known organic cofactor, plays a key role as the last methyl carrier in all methanogens [2]. Thus, methane is formed upon the reduction of methyl-CoM with coenzyme B (CoB) as an electron donor by the methyl-CoM reductase. The oxidation of CoB yields a heterodisulfide with CoM (CoM-S-S-CoB), which is reduced to regenerate the thiols by heterodisulfide reductase (Hdr) [3]. Without coenzyme M being present to complete the biosynthesis of methane, the organism is unable to produce the necessary energy for growth.

 The biosynthetic pathway of coenzyme M (CoM) in *Methanocaldococcus jannaschii*, an organism closely related to *M. maripaludis*, is proposed to proceed in five steps. Four enzymes involved in the biosynthesis of CoM have been biochemically characterized [7–10]. In addition, the genes encoding these enzymes have been identified in diverse methanogens, including *M. maripaludis*. The proposed pathway for the biosynthesis of CoM starts with the sulfonation of PEP by a phosphoenolpyruvate sulfotransferase (ComA). Then, a phosphosulfolactate phosphatase (ComB) hydrolyses phosphosulfolactate, and a dehydrogenase (ComC) oxidizes the (*R*)-sulfolactate intermediate to form sulfopyruvate. In the fourth step, a sulfopyruvate decarboxylase (ComDE) catalyzes the decarboxylation of sulfopyruvate to form sulfoacetaldehyde [10]. For the final postulated step of CoM biosynthesis, sulfoacetaldehyde is reductively thiolated to form coenzyme M. However, this enzyme has not yet been identified. In addition, the genomes of *Methanosarcina* species lack the genes for the first three steps of the pathway, suggesting that an alternative pathway for CoM biosynthesis exists [2]. Lastly, none of the steps of this pathway have been confirmed *in vivo* by mutagenesis of the proposed genes.

*M. maripaludis* may have the ability to take up coenzyme M from the medium. Previous studies have identified an energy-dependent transport system for coenzyme M within *Methanococcus voltae *[11]. Because these methanococci share many metabolic and physiological features, we hypothesized that the same uptake system is present in *M. maripaludis*. In addition, coenzyme M auxotrophs were also isolated by random mutagenesis in *M. voltae*, but the genetic loci of the mutations were not identified [12].

 In the present study, coenzyme M auxotrophs for the methanogenic archaeon *M. maripaludis* were made by *in vitro* transposon mutagenesis followed by transformation into the genome. This system was previously used to construct tryptophan auxotrophs in *M. maripaludis *[6]. The selected target was the gene *comE* (MMP1689), which encodes one of the subunits of the enzyme that catalyzes the decarboxylation of sulfopyruvate to sulfoacetaldehyde. In minimal medium in the absence of coenzyme M, the mutant grew poorly, and normal growth was restored by the addition of coenzyme M. Thus, coenzyme M stimulated but was not absolutely required for growth. These results confirmed the role of ComE in coenzyme M biosynthesis and the ability of *M. maripaludis* to take up CoM.

## 2. Materials and Methods

### 2.1. Strains, Primers, and Plasmids

Strains, primers, and plasmids are summarized in Table 1. All primers made in this work were designed using the Primer3Plus software [13].

**Figure 1:**
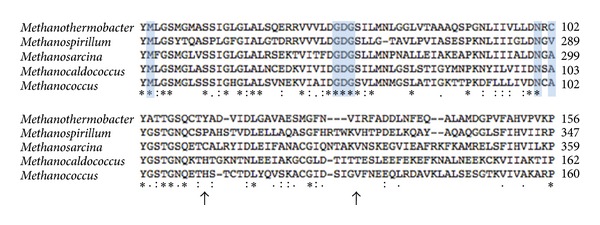
Alignment of partial amino acid sequences of the enzyme sulfopyruvate decarboxylase from methanoarchaea, from top to bottom: *Methanothermobacter thermautotrophicus, Methanospirillum hungatei, Methanosarcina acetivorans, Methanocaldococcus jannaschii*, and *Methanococcus maripaludis*. The arrows signify insertion positions of the Tn5〈KAN-2-pac〉 transposon into the gene *comE* (558 bp) of *M. maripaludis*. The insertion at 336 bp (112 aa) corresponds to the mutation in strain *M. maripaludis* S201, and the insertion at 395 bp (131 aa) corresponds to the mutation in strain *M. maripaludis* S202. Blue boxes containing aligned amino acids correspond to the conserved thiamine pyrophosphate-binding domain. Asterisks, double dots, and single dots denote positions that contain fully conserved amino acid residues, groups of strongly similar residues, and groups of weakly similar residues, respectively.

 For protein sequence alignment of the enzyme sulfopyruvate decarboxylase between methanogen species, ClustalW2 software from the European Bioinformatics Institute was used (Figure 1). Species consisted of *Methanothermobacter thermautotrophicus* (NCBI reference sequence O27275)*, Methanospirillum hungatei *(NCBI reference sequence YP504382),* Methanosarcina acetivorans *(NCBI reference sequence AAM06668)*, Methanocaldococcus jannaschii* (NCBI reference sequence P58416), and* Methanococcus maripaludis *(NCBI reference sequence NP988809).

### 2.2. Growth Conditions

*E. coli *was grown in Luria-Bertani medium at 37°C. For solid medium preparation, 1% agar was added. Ampicillin (100 *μ*g/mL) and kanamycin (50 *μ*g/mL) were supplemented when indicated.

*Methanococcus maripaludis* strain S2 was grown in 28 mL Balch tubes pressurized to 275 kPa with H_2_/CO_2_ (80 : 20, v/v) at 37°C in 5 mL of minimal (McN) or complex (McCV) medium reduced with 3 mM cysteine in anaerobic conditions as described previously [5]. When indicated, McCV was supplemented with 3 mM CoM to form McCm medium, and McN was supplemented with 10 mM acetate to form McNA. Before inoculation, 3 mM sodium sulfide was added. Puromycin (2.5 *μ*g/mL) and neomycin (500 *μ*g/mL) were added when indicated. For solid medium preparation, 1% agar was added. 

 Dry weight of the cultures was calculated assuming a conversion factor of 340 mg dry weight (L)^−1^ for a culture with an OD_600_ = 1.0 [14].

 To avoid residual coenzyme M contamination, the glassware used in this study was heated for 4-5 hours at 180°C. For some experiments, the glassware was heated for 24 hours at 425°C.

### 2.3. Construction of* comE*::Tn5 Mutant

PCR amplification of the *comE* genetic region was performed using primers RegcomEF and RegcomER (Table 1) and *Taq *DNA Polymerase (Fermentas). The cycling program proceeded as follows. After 3 min of denaturation at 95°C, the following steps were performed for 30 cycles: denaturation at 95°C for 30 s, annealing at 58°C for 3 min, and extension at 72°C for 3 min. A final extension was performed at 72°C for 10 min. The 3089 bp amplification product and the plasmid vector pUC18 were digested using restriction enzymes BamHI and XbaI at 37°C for 90 min and ligated together using T4 ligase (New England Biolab) at 25°C for 30 min. The resulting plasmid, pComE (5775 bp), was transformed into Transformax EC100 electrocompetent cells (Epicentre) by electroporation in a 2 mm electrode gap cuvette (2.36 kV). Samples were resuspended in 1 mL of LB medium, incubated for 1 hour at 37°C, and spread on LB agar plates in the presence and absence of ampicillin (100 *μ*g/mL) to determine transformation efficiency. Clones were picked, and plasmid was extracted using a Zyppy Plasmid miniprep Kit (Zymo Research). For screening, 1 *μ*g of plasmid was digested using the following sets of restriction enzymes (Sph1/Nde1/Alwn1 and Kpn1/Nco1 (New England Biolab)). 

 The Tn5〈KAN-2-pac〉 transposon [6] was PCR amplified using 5′ phosphorylated oligonucleotides ME-Plus9-3′ and ME-Plus9-5′ (Epicenter) and Phusion high-fidelity DNA polymerase (New England Biolabs). The cycling program proceeded as follows. After 3 min of denaturation at 98°C, the following steps were performed for 30 cycles: denaturation at 98°C for 30 s, annealing at 55°C for 60 s, and extension at 72°C for 140 s. A final extension was performed at 72°C for 10 min. The PCR product was purified using the DNA clean and concentrator (-5) kit (Zymo Research) and resuspended in TE buffer (10 mM Tris-HCl buffer, pH 7.5, 1 mM EDTA). *In vitro* transposition was achieved by incubation of 80 ng of the transposon, 200 ng of pComE, and 1 *μ*L of EZ-Tn5 transposase (1 U/*μ*L, Epicentre) following the manufacturer instructions (Epicentre). The mixture was transformed into Transformax EC100 electrocompetent cells (Epicentre) following the manufacturer instructions. Dilutions of the mixture were spread onto plates containing ampicillin (100 *μ*g/mL) or kanamycin (50 *μ*g/mL). Isolated kanamycin-resistant colonies were picked and grown in broth in the presence of both ampicillin and kanamycin. Plasmids with the transposon insertions were extracted using a Zyppy Plasmid miniprep Kit (Zymo Research). For screening, insertions were sequenced from the end of the transposon using the primer KAN-2RP-1out2 [6] at the University of Michigan DNA Sequencing Core. Two plasmids with different insertions in the *comE* gene were found and named pComET1 and pComET2.

Transformation into *M. maripaludis* with circular plasmids pComET1 and pComET2 was performed as described previously [5]. The transformation mixture was serially diluted, and 500 *μ*L was spread onto McCm agar plates supplemented with puromycin. After incubation for 6 days in the presence of ~150 kPa of H_2_/CO_2_ (80 : 20, v/v) at 37°C, isolated puromycin-resistant colonies were picked and restreaked onto McCm agar plates supplemented with puromycin. After growth under the same conditions, isolated puromycin-resistant colonies were transferred to stoppered culture tubes containing 5 mL of McCm medium plus puromycin, pressurized to 275 kPa, and incubated at 37°C. Frozen stocks were prepared in McCm medium + 30% glycerol (v/v), and the suspensions were stored at −80°C. The new strains with the integrated plasmids pComET1 and pComET2 were named *M. maripaludis* S201 and S202, respectively (Table 1). To further purify the mutant S201, frozen stocks were inoculated in 5 mL of McCm plus puromycin, pressurized to 275 kPa of H_2_/CO_2_ (80 : 20, v/v), and incubated at 37°C. The cell suspension was serially diluted and plated in serum-bottle agar slants with McCm medium plus puromycin [5]. After 5 days of incubation in the presence of 137 kPa of H_2_/CO_2_ (80 : 20, v/v) at 37°C, isolated puromycin-resistant colonies were picked and transferred to stoppered culture tubes containing 5 mL of McCm medium plus puromycin. Frozen stocks were prepared, and the suspensions were stored at −80°C. Genomic DNA was extracted using ZR Fungal/Bacterial DNA MiniPrep (Zymo Research).

Verification of the genotype of the mutant was determined through PCR using two sets of primers (Table 1). The first set consisted of a forward primer (KAN-2RP-1out2) from the end of the transposon and a reverse primer from the surrounding gene MMP1688 (MMP1688R) and was used to demonstrate the transposon insertion. The second set consisted of forward primer comEF and reverse primer comER, which amplified the wild type *comE* gene. The products were PCR amplified using Phusion high-fidelity DNA polymerase (New England Biolabs). The cycling program for both sets of primers proceeded as follows. After 1 min of denaturation at 98°C, the following steps were performed for 30 cycles: denaturation at 98°C for 15 s, annealing at 59°C for 30 s, and extension at 72°C for 30 s. A final extension was performed at 72°C for 10 min. The PCR products were electrophoresed on a 1% agarose gel and stained with ethidium bromide.

To determine the phenotype of the mutant, ~1 × 10^5^ mutant or wild type S2 cells were inoculated in 5 mL of McNA in the presence or the absence of 146 *μ*M of coenzyme M. The cultures were grown in 28 mL Balch tubes pressurized to 275 kPa with H_2_/CO_2_ for 90 hours in the absence of antibiotic. The second and third passages were performed by inoculating ~1 × 10^4^ mutant or wild type S2 cells from the previous cell suspensions into 5 mL of McNA in the presence or the absence of 146 *μ*M of coenzyme M and incubated under the same conditions for 100 and 480 hours, respectively. 

 For complementation, the *comE *gene was PCR amplified from the genomic DNA of *M. maripaludis* strain S2 using primers comEexpF and comEexpR (Table 1). The PCR products and the shuttle vector pMEVII were digested with NsiI and BglII and gel purified. The cloning of the PCR products into the digested pMEVII replaced the beta-galactosidase gene, and the resulting plasmid, pMEVII-*comE*, was transformed into S201 using the PEG-mediated method described above without coenzyme M supplemented to the medium. The transformation culture was screened on McCV plates containing neomycin and puromycin, and the complementation strain was named S203. The phenotype of the mutant S203 was determined by inoculating ~1 × 10^4^ mutant cells in the presence and the absence of puromycin + neomycin.

## 3. Results

**Figure 2:**
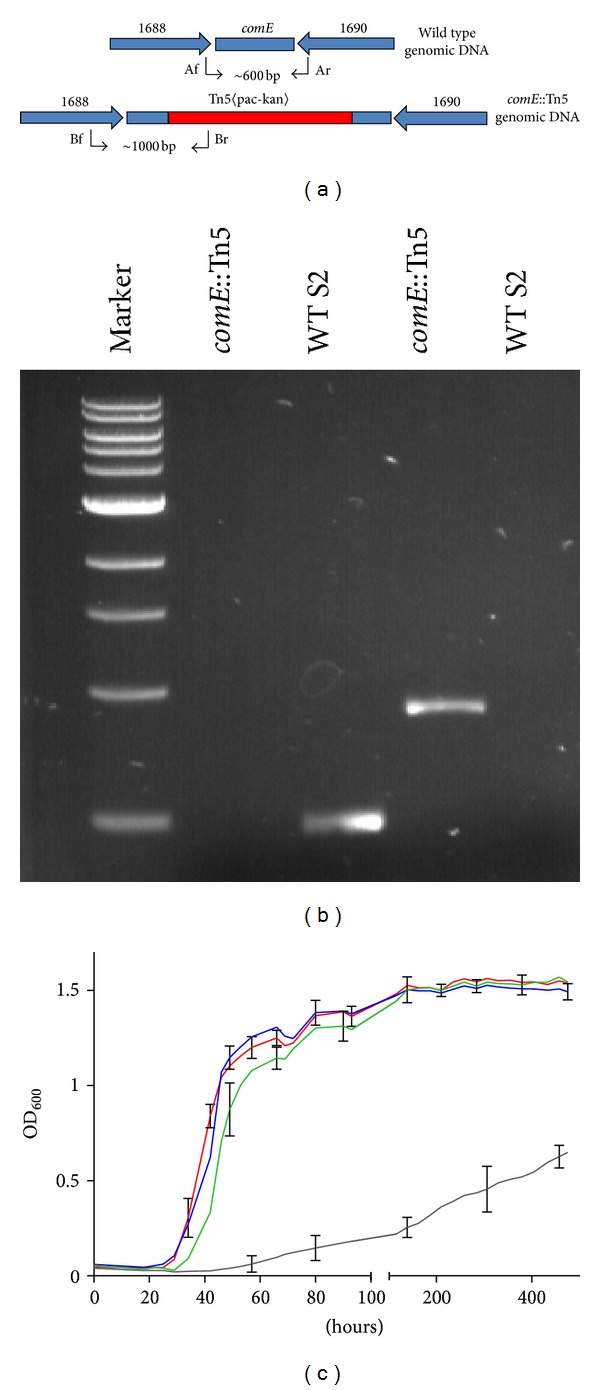
Characterization of the *comE*::Tn5 mutant strain S201. (a) Genetic maps of the gene *comE* in the wild type strain and the *comE*::Tn5 mutant strain S201. Numbers indicate the MMP identification, and the black arrows indicate primers used for PCR amplification. (b) Genotypic characterization of the *comE*::Tn5 mutant strain 201 by PCR amplification. Lane 1, standard 1 kb ladder (New England Biolab); Lanes 2 and 3, PCR amplifications of *comE* using primers comEF (Af) and comER (Ar) for genomic DNA of the *comE*::Tn5 mutant strain S201 and wild type strain S2, respectively; Lanes 4 and 5, PCR amplifications using a primer from the end of the transposon (KAN-2RP-1out2; Br) and a primer that binds upstream of the gene *comE* (Bf) for the same DNAs. (c) Growth of the wild type and *comE*::Tn5 mutant strain 201 in minimal medium + acetate (McNA) and McNA supplemented with coenzyme M after three passages. Grey, *comE*::Tn5 McNA; green, *comE*::Tn5 McNA + CoM; red, S2 McNA; blue, S2 McNA + CoM. Representative error bars indicate the standard deviation of three independent cultures. The inoculum was ~1 × 10^4^ cells.

The gene encoding ComE was disrupted by *in vitro* transposon mutagenesis with the Tn5〈KAN-2-pac〉 transposon [6], which encodes puromycin resistance in methanococci. In the first step, the transposon was randomly inserted in a plasmid containing a 3089 bp chromosomal region of *M. maripaludis* harboring the *comE* gene. Two independent transposon insertions in the *comE* gene were mapped downstream of the thiamine pyrophosphate binding domain (Figure 1). The wild type *comE* gene was replaced in the genome by the transposon-disrupted genes through homologous recombination, generating strains S201 and S202, with insertions at locations 336 bp and 395 bp from the 5′ start of the gene, respectively (Figure 1). Because both insertions were near each other, only one of the strains (S201) was selected for further characterization. The genotype of the mutant was confirmed by two independent PCR amplifications (Figure 2). When using primers to amplify the *comE *gene, only the wild type strain displayed a band at approximately 600 bp. Under these conditions, the mutant did not yield an amplification product, presumably because the length and high GC content of the *pac* cassette inside the transposon prevented amplification. In contrast, using a primer from the end of the transposon into the *comE *gene, a product of the expected size was found with the mutant but not the wild type strain. 

 When grown in minimal medium plus acetate in the absence of coenzyme M, the mutant strain S201 displayed a longer lag phase of between 80 and 120 h, slower growth rate during exponential phase, and lower maximum growth than the wild type during the time of the experiments. This “sick” phenotype was restored by the addition of 146 *μ*M coenzyme M. These results were observed even after three passages with a carryover of coenzyme M of 1.2 pM, much less than the 70 nM concentration required for growth of *M. voltae *(Figure 2(c)) [12]. Under these conditions, the doubling time (means ± standard deviations of three cultures) of S2 in McNA medium in the absence of coenzyme M and S201 in McNA medium in the absence and presence of coenzyme M was 4.9 ± 0.4, 162 ± 2, and 5.9 ± 0.4 hours, respectively. The maximum growth observed (means ± standard deviations of three cultures) of S2 in McNA medium in the absence of coenzyme M and S201 in McNA medium in the absence and presence of coenzyme M was 0.53 ± 0.01, 0.22 ± 0.03, and 0.53 ± 0.02 mg dry weight mL^−1^ of culture, respectively. Assuming a coenzyme M content of 0.43 nmol (mg dry weight)^−1^ [15], the minimum rates of CoM biosynthesis necessary to support the observed growth rates were 0.06 and 0.002 nmol (mg dry weight h)^−1^ for S2 and S201, respectively, indicating that the mutant retained no more than 3% of the wild type level of coenzyme M production. 

 Because only low levels were required for growth, it is possible that CoM contamination of the glassware was responsible for the observed growth. To test this possibility, different times and temperatures for heat-treating glassware were evaluated (data not shown). After heat-treating clean glassware and glassware intentionally “contaminated” with CoM at 425°C, a temperature previously demonstrated to eliminate small traces of CoM [16], the doubling time for the mutant strain S201 (means ± standard deviations of three cultures) was 160 ± 17 and 165 ± 13 hours, which was similar to the growth rate of the mutant strain S201 on the third passage in glassware heat-treated for 4 hours at 180°C. Thus, the low amount of growth of the mutant was not the result of small amounts of contaminating CoM in the medium. 

**Figure 3:**
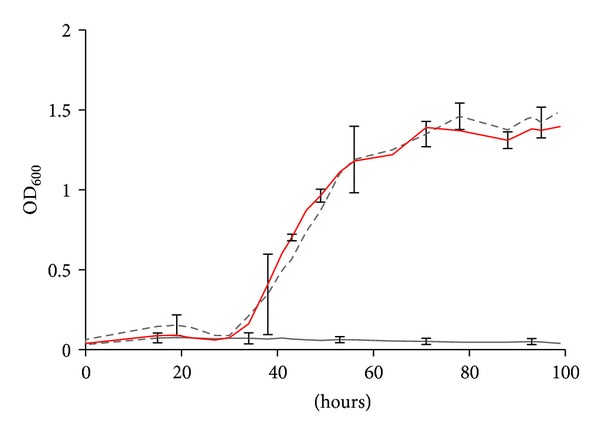
Growth of the wild type strain S2 (red), *comE*::Tn5 mutant strain S201 (grey), and complemented strain S203 with *comE* gene expressed from pMEVII-*comE* (dash grey) in minimal medium + acetate (McNA). Representative error bars indicate the standard deviation of three independent cultures. The inoculum was ~1 × 10^4^ cells.

 To confirm that the differences in maximum growth and growth rate were due to the transposon insertion, the complementation strain S203 was constructed by transforming the shuttle vector pMEVII-*comE* into S201, where the gene *comE* was expressed by the *M. voltae *histone promoter (PhmvA). The *trans* expression of the gene *comE* fully restored the growth of the mutant (Figure 3). 

**Figure 4:**
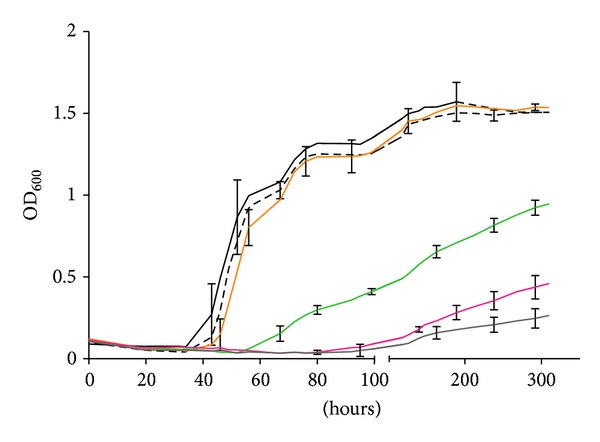
Growth of the *comE*::Tn5 mutant strain S201 in response to varying concentrations of CoM. Cultures were grown in McNA after three transfers in medium without CoM. Grey, no added CoM; red, + 0.5 nM CoM; green, + 10 nM CoM; orange, + 250 nM CoM; dash black, + 1.4 *μ*M CoM; black, + 146 *μ*M CoM. Representative error bars indicate the standard deviation of three independent replicate cultures. The inoculum was ~1 × 10^4^ cells from the second transfer.

 The growth of the mutant strain S201 was tested in the presence of different concentrations of CoM (Figure 4). The doubling times of the mutant strain S201 in McNA medium supplemented with 146 *μ*M, 1.4 *μ*M, 250 nM, 10 nM, 0.5 nM, and the absence of coenzyme M were 5.3 ± 0.3, 5.3 ± 0.5, 7.2 ± 1.0, 77 ± 3, 90 ± 11, and 153 ± 16 hours, respectively. Concentrations of coenzyme M over 1.4 *μ*M fully restored wild type growth rates. In contrast, when the concentrations of coenzyme M were 10 nM or less, the mutant grew poorly. Thus, growth of the mutant was severely impaired in the absence of CoM, but CoM was not essential for growth. This conclusion is supported by the increase in yields when the medium was supplemented with small amounts of CoM. Based upon the CoM levels measured in whole cells of 0.43 nmol/mg dry weight [15], the expected maximum growth in the presence of 10 nM CoM was 0.023 mg dry weight mL^−1^. However, the observed increase in growth was 0.32 ± 0.01 mg dry weight mL^−1^ or 14 times higher (Figure 4). These results confirmed that the mutant was capable of low levels of CoM biosynthesis.

## 4. Discussion

A partial auxotroph for coenzyme M was constructed in *M. maripaludis* through the disruption of the gene *comE*, which encodes the sulfopyruvate decarboxylase *β*-subunit involved in the fourth step of the proposed pathway of CoM biosynthesis [2]. The mutant exhibits impaired growth in minimal medium + acetate, which is fully restored upon the addition of coenzyme M, indicating that ComE is important for the biosynthesis of CoM. These results confirm that ComE plays a major role in CoM biosynthesis in *M. maripaludis*. 

 However, the transposon insertion of *comE* did not yield a complete CoM auxotroph, suggesting that *M. maripaludis* still possesses a source of coenzyme M. The manner that coenzyme M is still produced is not known. However, three possibilities are likely. The thiamine pyrophosphate-binding domain important to the decarboxylation activity and most of the conserved amino acid residues were upstream of the transposon insertion. Thus, it is possible that the ComE produced after mutagenesis still retained sufficient activity to support slow growth. Alternatively, a promiscuous enzyme might have replaced the sulfopyruvate decarboxylase in the CoM biosynthetic pathway. The genome of *M. maripaludis* S2 encodes the protein MMP0142, which possesses 28% sequence similarity in a 120 aa region of overlap with ComE. Like ComE, this enzyme belongs to the thiamine pyrophosphate (TPP) superfamily and exhibits a pyrimidine (PYR) binding domain. Possibly, MMP0142 is involved in a different biosynthetic pathway but possesses some fortuitous affinity with sulfopyruvate. A final possibility is that *M. maripaludis* possesses an unknown alternative pathway for coenzyme M. Nevertheless, to solve this enigma more experiments have to be performed. 

## Figures and Tables

**Table 1:** List of strains, plasmids, and primers.

Strains	Description	Source
*M. maripaludis* S2	Wild type	[4]
*M. maripaludis *S201	*comE::*Tn5<KAN-2-pac>	This work
*M. maripaludis *S202 *M. maripaludis* S203	*comE::*Tn5<KAN-2-pac> Strain S201 complemented with pMEVII-*comE *	This work This work
*E. coli* EC100	F *mcrA* Δ(*mrr-hsdRMS mcrBC) *Φ*80dlacZ*Δ*M15* Δ*lacX74 recA1 endA1 araD139 *Δ(*ara, leu*)*7697 galU galK λ rpsI *(str)* nupG *	Epicentre

Plasmids		

pUC18	*E. coli *plasmid cloning vector	GenScript
pComE	Plasmid pUC18 containing the genetic region of gene *comE *	This work
PComET1 and 2	pComE containing Tn5 transposon insertions in *comE* gene	This work
pMEVI and pMEVII	Shuttle plasmids for *M. maripaludis *	[5]
pMEVII-*comE *	Plasmid pMEVII containing gene *comE* in *trans *	This work

Primers		

RegcomEF	5′-AAAAAAGGATCCCGGATCTGACCCATACAATAGAG	This work
RegcomER	5′-AAAAAATCTAGAATGGATGGATTG GCAGTTTC	This work
ME-Plus9-3′	5′-CTGTCTCTTATACACATCTCAACCATCA	Epicentre
ME-Plus9-5′	5′-CTGTCTCTTATACACATCTCAACCCTGA	Epicentre
KAN-2RP-1out2	5′-GCAATGTAACATCAGAGATTTTGAC	[6]
MMP1688R	5′-CGAATGGATTCTTTTGAACTTTT	This work
comEF	5′-TTGCGTTCATAAATCTGTGTTT	This work
comER comEexpF comEexpR	5′-AATGGAATACGTGACCGATG 5′-ATATATATGCATGGAACTTACGAGATA 5′-ATATATAGATCTTTTATTTTTTTATTGCG	This work This work This work
M13R	5′-CAGGAAACAGCTATGACC	
